# Supplementary material for: Single Cell Transcriptomic and Chromatin Profiles Suggest Layer Vb Is the Only Layer With Shared Excitatory Cell Types in the Medial and Lateral Entorhinal Cortex
Source: Front Neural Circuits. 2022 Jan 26;15:806154. doi: 10.3389/fncir.2021.806154 (PMC8826650; doi:10.3389/fncir.2021.806154)
Supplement: Supplementary file 14 [file Data_Sheet_1.ZIP › 17/knownResults.html]

17 - Homer Known Motif Enrichment Results


# Homer Known Motif Enrichment Results (17)

Homer *de novo* Motif Results  
Gene Ontology Enrichment Results  
Known Motif Enrichment Results (txt file)  
Total Target Sequences = 1000, Total Background Sequences = 47826

|  |  |  |  |  |  |  |  |  |  |  |  |
| --- | --- | --- | --- | --- | --- | --- | --- | --- | --- | --- | --- |
| Rank | Motif | Name | P-value | log P-pvalue | q-value (Benjamini) | # Target Sequences with Motif | % of Targets Sequences with Motif | # Background Sequences with Motif | % of Background Sequences with Motif | Motif File | SVG |
| 1 | A G T C A T C G C T A G A G C T G A C T C T A G A G T C A G T C G C T A C A G T T C A G T C A G G A T C C T G A T C G A G A T C | RFX(HTH)/K562-RFX3-ChIP-Seq(SRA012198)/Homer | 1e-26 | -6.121e+01 | 0.0000 | 56.0 | 5.60% | 411.3 | 0.86% | motif file (matrix) | svg |
| 2 | C T A G A G C T G A C T C A T G A G T C A G T C G T C A C A G T C T A G T C A G G T A C C T G A T C G A G A T C T G A C | Rfx2(HTH)/LoVo-RFX2-ChIP-Seq(GSE49402)/Homer | 1e-24 | -5.556e+01 | 0.0000 | 57.0 | 5.70% | 481.0 | 1.01% | motif file (matrix) | svg |
| 3 | A G C T A C G T A C T G A T G C A G T C C G T A C T G A T A C G | NF1-halfsite(CTF)/LNCaP-NF1-ChIP-Seq(Unpublished)/Homer | 1e-23 | -5.297e+01 | 0.0000 | 367.0 | 36.70% | 10790.2 | 22.68% | motif file (matrix) | svg |
| 4 | T C G A A C G T A C G T C T G A G A T C T C A G G A C T G T C A C G T A A G C T G T C A C T A G A G C T A C G T T C G A | NFIL3(bZIP)/HepG2-NFIL3-ChIP-Seq(Encode)/Homer | 1e-17 | -4.058e+01 | 0.0000 | 176.0 | 17.60% | 4214.1 | 8.86% | motif file (matrix) | svg |
| 5 | T C G A A C G T C A T G G C T A T A G C C G A T G T A C G C T A A C G T A T G C | AP-1(bZIP)/ThioMac-PU.1-ChIP-Seq(GSE21512)/Homer | 1e-17 | -3.940e+01 | 0.0000 | 181.0 | 18.10% | 4441.2 | 9.33% | motif file (matrix) | svg |
| 6 | C A G T T G C A A C G T A C T G C G T A A T C G C G A T T G A C C G T A A C G T | BATF(bZIP)/Th17-BATF-ChIP-Seq(GSE39756)/Homer | 1e-16 | -3.796e+01 | 0.0000 | 168.0 | 16.80% | 4052.3 | 8.52% | motif file (matrix) | svg |
| 7 | C T A G T C G A A C G T A C T G C G T A A T G C A C G T G T A C C G T A A G C T G A T C G T A C | Atf3(bZIP)/GBM-ATF3-ChIP-Seq(GSE33912)/Homer | 1e-16 | -3.795e+01 | 0.0000 | 166.0 | 16.60% | 3983.0 | 8.37% | motif file (matrix) | svg |
| 8 | C A T G C T A G A G C T G A C T C A T G A G T C G A T C G C T A C G A T C T A G T C A G G T A C C T G A T C G A | X-box(HTH)/NPC-H3K4me1-ChIP-Seq(GSE16256)/Homer | 1e-16 | -3.772e+01 | 0.0000 | 54.0 | 5.40% | 645.0 | 1.36% | motif file (matrix) | svg |
| 9 | C T A G T C G A G C A T C A T G G C T A T A G C C G A T G T A C C T G A A G C T | JunB(bZIP)/DendriticCells-Junb-ChIP-Seq(GSE36099)/Homer | 1e-15 | -3.667e+01 | 0.0000 | 143.0 | 14.30% | 3255.6 | 6.84% | motif file (matrix) | svg |
| 10 | A C T G C A T G T C G A A C G T A C T G C G T A A T C G A C G T G T A C C G T A G A C T A G T C | Fos(bZIP)/TSC-Fos-ChIP-Seq(GSE110950)/Homer | 1e-15 | -3.583e+01 | 0.0000 | 152.0 | 15.20% | 3594.3 | 7.55% | motif file (matrix) | svg |
| 11 | C A T G C T A G T C G A A C G T A C T G C G T A T A G C C G A T T G A C C G T A A G C T G A T C | Fra2(bZIP)/Striatum-Fra2-ChIP-Seq(GSE43429)/Homer | 1e-14 | -3.370e+01 | 0.0000 | 124.0 | 12.40% | 2744.9 | 5.77% | motif file (matrix) | svg |
| 12 | C T A G T C G A A C G T A C T G C G T A T A G C C G A T G T A C C G T A A G C T G A T C G T A C | Jun-AP1(bZIP)/K562-cJun-ChIP-Seq(GSE31477)/Homer | 1e-14 | -3.347e+01 | 0.0000 | 75.0 | 7.50% | 1259.5 | 2.65% | motif file (matrix) | svg |
| 13 | C T A G T C G A C G A T A C T G C G T A T A C G A G C T T G A C G C T A A C G T G A T C T A G C | Fosl2(bZIP)/3T3L1-Fosl2-ChIP-Seq(GSE56872)/Homer | 1e-14 | -3.312e+01 | 0.0000 | 93.0 | 9.30% | 1789.0 | 3.76% | motif file (matrix) | svg |
| 14 | T C A G G A C T C A G T C T G A A G C T C T A G G A C T T G C A C T G A A G T C | HLF(bZIP)/HSC-HLF.Flag-ChIP-Seq(GSE69817)/Homer | 1e-14 | -3.274e+01 | 0.0000 | 213.0 | 21.30% | 5954.9 | 12.51% | motif file (matrix) | svg |
| 15 | A C T G C T A G T C G A C G A T C A T G G C T A A T C G C G A T G T A C G C T A A G C T G T A C | Fra1(bZIP)/BT549-Fra1-ChIP-Seq(GSE46166)/Homer | 1e-14 | -3.233e+01 | 0.0000 | 140.0 | 14.00% | 3336.8 | 7.01% | motif file (matrix) | svg |
| 16 | A G T C G A C T C A G T A C T G C T A G T G A C G C T A A T G C G C A T A T C G C G A T A C T G G A T C G T A C G T C A C T G A | NF1(CTF)/LNCAP-NF1-ChIP-Seq(Unpublished)/Homer | 1e-13 | -3.160e+01 | 0.0000 | 95.0 | 9.50% | 1898.3 | 3.99% | motif file (matrix) | svg |
| 17 | A G C T C A T G G C A T G A T C T G C A C T A G G A T C A C G T | Tgif2(Homeobox)/mES-Tgif2-ChIP-Seq(GSE55404)/Homer | 1e-12 | -2.984e+01 | 0.0000 | 564.0 | 56.40% | 21305.0 | 44.77% | motif file (matrix) | svg |
| 18 | A G T C C G A T A C T G A T C G T G A C G C T A C A T G A T C G T G A C C G A T A C T G T A G C G T A C G T C A | Tlx?(NR)/NPC-H3K4me1-ChIP-Seq(GSE16256)/Homer | 1e-12 | -2.783e+01 | 0.0000 | 106.0 | 10.60% | 2384.6 | 5.01% | motif file (matrix) | svg |
| 19 | A C T G T C A G A G C T G A C T C A T G A G T C A G T C G C T A C G A T C T A G T C A G G T A C C T G A T C G A | Rfx1(HTH)/NPC-H3K4me1-ChIP-Seq(GSE16256)/Homer | 1e-12 | -2.779e+01 | 0.0000 | 65.0 | 6.50% | 1126.1 | 2.37% | motif file (matrix) | svg |
| 20 | G A C T G C A T C T A G C G A T G A T C T C G A C A T G G A T C | Tgif1(Homeobox)/mES-Tgif1-ChIP-Seq(GSE55404)/Homer | 1e-7 | -1.652e+01 | 0.0000 | 512.0 | 51.20% | 20390.0 | 42.85% | motif file (matrix) | svg |
| 21 | G A C T C T A G A T G C A G T C G T C A T A C G A T G C A T C G | HIC1(Zf)/Treg-ZBTB29-ChIP-Seq(GSE99889)/Homer | 1e-6 | -1.527e+01 | 0.0000 | 339.0 | 33.90% | 12672.8 | 26.63% | motif file (matrix) | svg |
| 22 | C T G A C T G A C A T G A T C G A G C T A T C G G A C T C A T G C T G A G T C A | Tbr1(T-box)/Cortex-Tbr1-ChIP-Seq(GSE71384)/Homer | 1e-6 | -1.499e+01 | 0.0000 | 255.0 | 25.50% | 9056.5 | 19.03% | motif file (matrix) | svg |
| 23 | C T G A T C A G G T A C G C T A A C T G T G A C G C A T C A T G | SCL(bHLH)/HPC7-Scl-ChIP-Seq(GSE13511)/Homer | 1e-6 | -1.419e+01 | 0.0000 | 657.0 | 65.70% | 27690.9 | 58.19% | motif file (matrix) | svg |
| 24 | C T G A C T A G A C T G G C A T C T A G G C A T A T C G C T G A C G T A G T C A | Tbx21(T-box)/GM12878-TBX21-ChIP-Seq(Encode)/Homer | 1e-5 | -1.379e+01 | 0.0000 | 185.0 | 18.50% | 6248.3 | 13.13% | motif file (matrix) | svg |
| 25 | G A C T C A G T A G C T C G A T A G T C G A T C A G T C C G T A A T G C T C A G | Rbpj1(?)/Panc1-Rbpj1-ChIP-Seq(GSE47459)/Homer | 1e-5 | -1.370e+01 | 0.0000 | 253.0 | 25.30% | 9116.7 | 19.16% | motif file (matrix) | svg |
| 26 | T G C A A G C T C T G A A T C G G A C T C T A G G T A C G A T C G T C A A G T C G T A C G A C T C T A G A T C G G C A T C A T G C A T G G A T C G T A C C T G A | CTCF(Zf)/CD4+-CTCF-ChIP-Seq(Barski\_et\_al.)/Homer | 1e-5 | -1.366e+01 | 0.0000 | 42.0 | 4.20% | 873.3 | 1.84% | motif file (matrix) | svg |
| 27 | T C A G A G C T A C G T A C G T G T A C G A T C C G T A C T A G C A T G G T C A C G T A T C G A | STAT4(Stat)/CD4-Stat4-ChIP-Seq(GSE22104)/Homer | 1e-5 | -1.299e+01 | 0.0000 | 191.0 | 19.10% | 6576.5 | 13.82% | motif file (matrix) | svg |
| 28 | C G A T T A C G T G A C G A C T C A T G C G T A T A C G A C G T G T A C C T G A | Bach2(bZIP)/OCILy7-Bach2-ChIP-Seq(GSE44420)/Homer | 1e-5 | -1.270e+01 | 0.0000 | 47.0 | 4.70% | 1069.6 | 2.25% | motif file (matrix) | svg |
| 29 | C A T G A G T C G A C T C G T A C G A T G C A T G A C T G C A T C G A T C T A G C A T G T G A C | Mef2b(MADS)/HEK293-Mef2b.V5-ChIP-Seq(GSE67450)/Homer | 1e-5 | -1.252e+01 | 0.0001 | 170.0 | 17.00% | 5757.3 | 12.10% | motif file (matrix) | svg |
| 30 | C T G A T G A C T G A C C G T A A C G T T G A C A G C T C T A G A C G T G A C T | Olig2(bHLH)/Neuron-Olig2-ChIP-Seq(GSE30882)/Homer | 1e-5 | -1.245e+01 | 0.0001 | 350.0 | 35.00% | 13537.5 | 28.45% | motif file (matrix) | svg |
| 31 | G T A C G A T C C A G T A G T C A G T C A G T C T G C A G A T C C T G A A T G C G T C A A C G T | WT1(Zf)/Kidney-WT1-ChIP-Seq(GSE90016)/Homer | 1e-5 | -1.182e+01 | 0.0001 | 103.0 | 10.30% | 3143.2 | 6.61% | motif file (matrix) | svg |
| 32 | G C T A G A C T G A C T T G C A C G T A A G T C C G T A T A G C G A T C G A C T | Eomes(T-box)/H9-Eomes-ChIP-Seq(GSE26097)/Homer | 1e-4 | -1.134e+01 | 0.0002 | 360.0 | 36.00% | 14150.0 | 29.74% | motif file (matrix) | svg |
| 33 | C G T A C T A G C A T G A G C T A C T G C G A T A T C G C G T A G T C A G T C A | Tbet(T-box)/CD8-Tbet-ChIP-Seq(GSE33802)/Homer | 1e-4 | -1.083e+01 | 0.0003 | 193.0 | 19.30% | 6898.4 | 14.50% | motif file (matrix) | svg |
| 34 | G T A C G A C T C G T A C T G A T C G A C G T A G C T A C A G T C T G A T A C G | Mef2a(MADS)/HL1-Mef2a.biotin-ChIP-Seq(GSE21529)/Homer | 1e-4 | -1.077e+01 | 0.0003 | 90.0 | 9.00% | 2723.8 | 5.72% | motif file (matrix) | svg |
| 35 | C T A G C T G A T C A G A T C G A G C T C A T G G A C T A G T C C T G A T G C A | Tbx6(T-box)/ESC-Tbx6-ChIP-Seq(GSE93524)/Homer | 1e-4 | -9.858e+00 | 0.0007 | 174.0 | 17.40% | 6211.6 | 13.05% | motif file (matrix) | svg |
| 36 | A T G C T C G A A G T C A G C T A C G T G T A C A G T C G C T A C T A G C A T G G T C A C T G A T C A G A G T C | Stat3+il21(Stat)/CD4-Stat3-ChIP-Seq(GSE19198)/Homer | 1e-4 | -9.850e+00 | 0.0007 | 134.0 | 13.40% | 4551.1 | 9.56% | motif file (matrix) | svg |
| 37 | C A G T T C A G G A T C A C T G A C G T C T A G A C T G A C T G G A C T C T A G | Egr1(Zf)/K562-Egr1-ChIP-Seq(GSE32465)/Homer | 1e-4 | -9.742e+00 | 0.0007 | 117.0 | 11.70% | 3872.3 | 8.14% | motif file (matrix) | svg |
| 38 | C A T G A G T C A G C T C G T A C G A T C G A T G C A T G C A T C G A T C T G A C A T G T G A C | Mef2d(MADS)/Retina-Mef2d-ChIP-Seq(GSE61391)/Homer | 1e-4 | -9.691e+00 | 0.0007 | 48.0 | 4.80% | 1243.5 | 2.61% | motif file (matrix) | svg |
| 39 | T G C A A G C T A C G T C T A G G A T C C T A G G A T C G T C A C T G A A G T C | CEBP(bZIP)/ThioMac-CEBPb-ChIP-Seq(GSE21512)/Homer | 1e-4 | -9.322e+00 | 0.0010 | 128.0 | 12.80% | 4360.9 | 9.16% | motif file (matrix) | svg |
| 40 | T C A G T G A C G T A C C G T A A C G T T G A C A C G T T C A G A G C T G A C T | NeuroD1(bHLH)/Islet-NeuroD1-ChIP-Seq(GSE30298)/Homer | 1e-3 | -9.068e+00 | 0.0013 | 147.0 | 14.70% | 5172.3 | 10.87% | motif file (matrix) | svg |
| 41 | C T G A C T A G A T C G A G C T A C T G G A C T A G T C C T G A | Tbx5(T-box)/HL1-Tbx5.biotin-ChIP-Seq(GSE21529)/Homer | 1e-3 | -8.852e+00 | 0.0015 | 465.0 | 46.50% | 19405.2 | 40.78% | motif file (matrix) | svg |
| 42 | T A C G T C G A G A C T A C T G C T G A A G T C T C A G G A C T T G A C C T G A | Atf1(bZIP)/K562-ATF1-ChIP-Seq(GSE31477)/Homer | 1e-3 | -8.575e+00 | 0.0020 | 117.0 | 11.70% | 3988.3 | 8.38% | motif file (matrix) | svg |
| 43 | A T G C A G T C G T A C A G C T T C G A C T A G G A T C C T G A G T C A A G T C G C T A T C A G | Rfx5(HTH)/GM12878-Rfx5-ChIP-Seq(GSE31477)/Homer | 1e-3 | -8.421e+00 | 0.0023 | 81.0 | 8.10% | 2561.9 | 5.38% | motif file (matrix) | svg |
| 44 | G A C T C G A T C T G A G T C A G A C T C G A T T C G A C G T A G C T A G C T A T G A C G T A C C G T A A C T G T G C A C G A T A C T G A C G T | Pitx1:Ebox(Homeobox,bHLH)/Hindlimb-Pitx1-ChIP-Seq(GSE41591)/Homer | 1e-3 | -8.035e+00 | 0.0032 | 49.0 | 4.90% | 1374.7 | 2.89% | motif file (matrix) | svg |
| 45 | A G T C A C G T A C T G A G C T A C G T A C G T G T C A A G T C | Foxo1(Forkhead)/RAW-Foxo1-ChIP-Seq(Fan\_et\_al.)/Homer | 1e-3 | -7.988e+00 | 0.0033 | 329.0 | 32.90% | 13302.5 | 27.96% | motif file (matrix) | svg |
| 46 | T G C A C G T A G T C A A G C T A G T C G C T A T A G C C G A T C T A G G A T C | Gfi1b(Zf)/HPC7-Gfi1b-ChIP-Seq(GSE22178)/Homer | 1e-3 | -7.577e+00 | 0.0049 | 126.0 | 12.60% | 4471.0 | 9.40% | motif file (matrix) | svg |
| 47 | A C G T C T A G C G T A A G T C G T A C A C G T A C G T A C G T G T C A G T A C T G A C G A C T | Nur77(NR)/K562-NR4A1-ChIP-Seq(GSE31363)/Homer | 1e-3 | -7.577e+00 | 0.0049 | 40.0 | 4.00% | 1076.3 | 2.26% | motif file (matrix) | svg |
| 48 | A T G C A G C T T C A G T G A C T C A G A T G C T G C A A C G T A T C G G A T C A C T G A G T C | NRF1(NRF)/MCF7-NRF1-ChIP-Seq(Unpublished)/Homer | 1e-3 | -7.492e+00 | 0.0051 | 36.0 | 3.60% | 939.3 | 1.97% | motif file (matrix) | svg |
| 49 | T C A G G C T A T C A G C A G T T G A C G T C A A G T C A T C G T G C A G T A C C A G T G A T C | Npas4(bHLH)/Neuron-Npas4-ChIP-Seq(GSE127793)/Homer | 1e-3 | -7.176e+00 | 0.0069 | 122.0 | 12.20% | 4351.1 | 9.14% | motif file (matrix) | svg |
| 50 | C T G A A C G T A C G T A C G T A G T C G A C T C G A T C T G A A C T G C G T A C G T A T C G A | STAT5(Stat)/mCD4+-Stat5-ChIP-Seq(GSE12346)/Homer | 1e-3 | -7.131e+00 | 0.0070 | 72.0 | 7.20% | 2319.9 | 4.88% | motif file (matrix) | svg |
| 51 | C T A G T C A G C A G T T C A G A C T G A C T G G A T C C T A G A C T G C T A G T C A G A T G C | KLF14(Zf)/HEK293-KLF14.GFP-ChIP-Seq(GSE58341)/Homer | 1e-3 | -6.972e+00 | 0.0081 | 236.0 | 23.60% | 9310.8 | 19.57% | motif file (matrix) | svg |
| 52 | T C A G A G C T A T G C C G T A A G T C T C A G A C G T A T C G T C G A A G T C G A T C T G A C | TFE3(bHLH)/MEF-TFE3-ChIP-Seq(GSE75757)/Homer | 1e-2 | -6.858e+00 | 0.0089 | 21.0 | 2.10% | 462.9 | 0.97% | motif file (matrix) | svg |
| 53 | T C A G A C G T T C G A T A G C A G T C C G T A A C T G G T A C A C G T A C T G A T C G A G T C | Atoh1(bHLH)/Cerebellum-Atoh1-ChIP-Seq(GSE22111)/Homer | 1e-2 | -6.845e+00 | 0.0089 | 182.0 | 18.20% | 6958.8 | 14.62% | motif file (matrix) | svg |
| 54 | G A C T C T A G G A T C C A G T A C T G C T G A A T G C G C A T A T G C C T G A | MafA(bZIP)/Islet-MafA-ChIP-Seq(GSE30298)/Homer | 1e-2 | -6.800e+00 | 0.0091 | 147.0 | 14.70% | 5456.5 | 11.47% | motif file (matrix) | svg |
| 55 | A T G C A T C G T A C G A G C T A T C G C T G A A G T C C T A G A G C T A T G C C T G A A T G C | CRE(bZIP)/Promoter/Homer | 1e-2 | -6.693e+00 | 0.0099 | 40.0 | 4.00% | 1129.6 | 2.37% | motif file (matrix) | svg |
| 56 | C T A G A C T G C T A G T C A G T C A G T A C G C T A G A C T G | Maz(Zf)/HepG2-Maz-ChIP-Seq(GSE31477)/Homer | 1e-2 | -6.598e+00 | 0.0107 | 175.0 | 17.50% | 6692.8 | 14.07% | motif file (matrix) | svg |
| 57 | T G A C A G T C C T G A T G A C C G T A A C G T A C G T A G T C A G T C C G T A | TEAD1(TEAD)/HepG2-TEAD1-ChIP-Seq(Encode)/Homer | 1e-2 | -6.437e+00 | 0.0124 | 175.0 | 17.50% | 6717.3 | 14.12% | motif file (matrix) | svg |
| 58 | T G C A A G C T C A T G C G T A A G C T A C T G G A T C G T C A C G T A A G C T | Atf4(bZIP)/MEF-Atf4-ChIP-Seq(GSE35681)/Homer | 1e-2 | -6.330e+00 | 0.0135 | 56.0 | 5.60% | 1762.0 | 3.70% | motif file (matrix) | svg |
| 59 | C A G T T C A G A G C T G A C T A C G T A G T C G A T C G A C T C T G A A C T G G A T C C G T A C T G A A G T C G T A C | Rfx6(HTH)/Min6b1-Rfx6.HA-ChIP-Seq(GSE62844)/Homer | 1e-2 | -6.192e+00 | 0.0153 | 178.0 | 17.80% | 6887.9 | 14.48% | motif file (matrix) | svg |
| 60 | A G T C G A C T C A G T G T A C A G T C A T C G T C A G A C T G G T C A C G T A | Stat3(Stat)/mES-Stat3-ChIP-Seq(GSE11431)/Homer | 1e-2 | -6.173e+00 | 0.0153 | 90.0 | 9.00% | 3141.9 | 6.60% | motif file (matrix) | svg |
| 61 | A G C T C T A G T G A C C G T A A C G T C G A T A G T C A G T C C T G A C A T G | TEAD3(TEA)/HepG2-TEAD3-ChIP-Seq(Encode)/Homer | 1e-2 | -6.133e+00 | 0.0156 | 204.0 | 20.40% | 8039.6 | 16.90% | motif file (matrix) | svg |
| 62 | C T G A T C A G C A G T C T A G A C T G C T A G G A T C A T C G A C T G C T G A T C A G G A T C | Sp5(Zf)/mES-Sp5.Flag-ChIP-Seq(GSE72989)/Homer | 1e-2 | -6.082e+00 | 0.0162 | 143.0 | 14.30% | 5387.1 | 11.32% | motif file (matrix) | svg |
| 63 | T A G C G T A C C G T A C T A G A C T G T G C A C G T A A T G C C G T A A T C G | AR-halfsite(NR)/LNCaP-AR-ChIP-Seq(GSE27824)/Homer | 1e-2 | -6.025e+00 | 0.0169 | 563.0 | 56.30% | 24650.6 | 51.81% | motif file (matrix) | svg |
| 64 | T A G C C T A G T C G A G A C T A C T G C T G A A G T C T C A G G C A T T G A C C T G A A G C T | Atf7(bZIP)/3T3L1-Atf7-ChIP-Seq(GSE56872)/Homer | 1e-2 | -6.013e+00 | 0.0169 | 79.0 | 7.90% | 2708.8 | 5.69% | motif file (matrix) | svg |
| 65 | T A C G G A C T T G A C C G T A A C G T G A T C G T C A C G T A A C G T A T G C C G T A G A C T | HOXA2(Homeobox)/mES-Hoxa2-ChIP-Seq(Donaldson\_et\_al.)/Homer | 1e-2 | -5.966e+00 | 0.0174 | 27.0 | 2.70% | 704.1 | 1.48% | motif file (matrix) | svg |
| 66 | T C G A T G A C G T A C C G T A C A G T T G A C A C G T A C T G A G C T A G C T | NeuroG2(bHLH)/Fibroblast-NeuroG2-ChIP-Seq(GSE75910)/Homer | 1e-2 | -5.964e+00 | 0.0174 | 257.0 | 25.70% | 10434.3 | 21.93% | motif file (matrix) | svg |
| 67 | T A G C A G T C T G A C A G T C C T A G A T C G A G T C C A T G T G A C A G T C G T A C A G T C A G T C G C A T C T A G A T C G G C A T A C T G A T C G G A T C | BORIS(Zf)/K562-CTCFL-ChIP-Seq(GSE32465)/Homer | 1e-2 | -5.814e+00 | 0.0196 | 46.0 | 4.60% | 1417.0 | 2.98% | motif file (matrix) | svg |
| 68 | A T G C T C A G T C G A G C A T A C T G C G T A A G T C T C A G G A C T T G A C C G T A A G C T | Atf2(bZIP)/3T3L1-Atf2-ChIP-Seq(GSE56872)/Homer | 1e-2 | -5.725e+00 | 0.0211 | 56.0 | 5.60% | 1813.0 | 3.81% | motif file (matrix) | svg |
| 69 | C G T A C G T A C G T A G C A T G C A T T A C G G T A C G A C T A C T G C G T A A T C G A C G T G T A C C G T A A G C T | Bach1(bZIP)/K562-Bach1-ChIP-Seq(GSE31477)/Homer | 1e-2 | -5.704e+00 | 0.0212 | 16.0 | 1.60% | 346.6 | 0.73% | motif file (matrix) | svg |
| 70 | T G C A T C G A T C G A A C G T C A T G C G T A A G T C T C A G A C G T G T A C C G T A A G C T | CREB5(bZIP)/LNCaP-CREB5.V5-ChIP-Seq(GSE137775)/Homer | 1e-2 | -5.645e+00 | 0.0222 | 68.0 | 6.80% | 2300.6 | 4.83% | motif file (matrix) | svg |
| 71 | G A C T C A G T G A T C G A T C A C G T G A T C C T G A T A C G C G T A G T C A | STAT6(Stat)/Macrophage-Stat6-ChIP-Seq(GSE38377)/Homer | 1e-2 | -5.482e+00 | 0.0258 | 102.0 | 10.20% | 3725.6 | 7.83% | motif file (matrix) | svg |
| 72 | T A C G C T A G A T G C G A T C G T A C A G T C C T A G A G T C A G T C A G T C G T A C A G T C | Sp1(Zf)/Promoter/Homer | 1e-2 | -5.397e+00 | 0.0277 | 51.0 | 5.10% | 1645.9 | 3.46% | motif file (matrix) | svg |
| 73 | A T G C C T G A G A C T A C G T A C G T G T A C G A T C C G A T C T A G C A T G C G T A C G T A C T G A G A C T | STAT1(Stat)/HelaS3-STAT1-ChIP-Seq(GSE12782)/Homer | 1e-2 | -5.395e+00 | 0.0277 | 61.0 | 6.10% | 2043.8 | 4.30% | motif file (matrix) | svg |
| 74 | C G T A G C T A C G A T C T A G A C G T G T C A C G T A C G T A A G T C C G T A T G C A T A C G | FoxL2(Forkhead)/Ovary-FoxL2-ChIP-Seq(GSE60858)/Homer | 1e-2 | -5.334e+00 | 0.0287 | 184.0 | 18.40% | 7298.5 | 15.34% | motif file (matrix) | svg |
| 75 | T A G C T C A G C A T G G C A T A G C T C G A T A T G C C G T A C G T A G T C A | CHR(?)/Hela-CellCycle-Expression/Homer | 1e-2 | -5.325e+00 | 0.0287 | 127.0 | 12.70% | 4812.4 | 10.11% | motif file (matrix) | svg |
| 76 | C A G T C G A T G C A T G C A T G T C A A G C T C A T G C T A G A T G C G T A C | Hoxa11(Homeobox)/ChickenMSG-Hoxa11.Flag-ChIP-Seq(GSE86088)/Homer | 1e-2 | -5.271e+00 | 0.0297 | 420.0 | 42.00% | 18077.6 | 37.99% | motif file (matrix) | svg |
| 77 | T C A G T A C G T A G C A C G T A C T G C G A T A G T C C G T A T A C G A G T C | Meis1(Homeobox)/MastCells-Meis1-ChIP-Seq(GSE48085)/Homer | 1e-2 | -5.187e+00 | 0.0319 | 261.0 | 26.10% | 10775.7 | 22.65% | motif file (matrix) | svg |
| 78 | C A T G A C T G C T A G T C G A T C G A T C G A T C G A T C A G T C A G T C A G T G A C T G A C C G T A A C T G T G C A C G A T A C T G | RBPJ:Ebox(?,bHLH)/Panc1-Rbpj1-ChIP-Seq(GSE47459)/Homer | 1e-2 | -4.920e+00 | 0.0412 | 56.0 | 5.60% | 1889.4 | 3.97% | motif file (matrix) | svg |
| 79 | T C A G T A G C G A C T C A T G C T G A A T C G G C A T G T A C C G T A A C T G T A G C T G C A | MafK(bZIP)/C2C12-MafK-ChIP-Seq(GSE36030)/Homer | 1e-2 | -4.879e+00 | 0.0424 | 48.0 | 4.80% | 1573.2 | 3.31% | motif file (matrix) | svg |
| 80 | A G C T G C A T A C T G A C G T A G T C A C G T C T A G T A C G | Smad3(MAD)/NPC-Smad3-ChIP-Seq(GSE36673)/Homer | 1e-2 | -4.809e+00 | 0.0449 | 440.0 | 44.00% | 19134.8 | 40.21% | motif file (matrix) | svg |
| 81 | A T C G A G C T A C T G A G T C A C T G A G T C C G T A A C G T A C T G A G T C A C T G A G T C | NRF(NRF)/Promoter/Homer | 1e-2 | -4.649e+00 | 0.0520 | 40.0 | 4.00% | 1278.6 | 2.69% | motif file (matrix) | svg |
| 82 | A T C G T G A C A T G C C T G A T C A G G A C T A G T C C G A T T C A G T C G A C A T G C T A G C T A G C G T A C T A G C T A G C T G A C T A G C T A G A T G C | ZSCAN22(Zf)/HEK293-ZSCAN22.GFP-ChIP-Seq(GSE58341)/Homer | 1e-2 | -4.648e+00 | 0.0520 | 14.0 | 1.40% | 322.1 | 0.68% | motif file (matrix) | svg |
| 83 | C T A G A T G C A T G C C G A T A C T G G A C T A T G C G C T A T G A C A G C T T A G C G C T A | PBX1(Homeobox)/MCF7-PBX1-ChIP-Seq(GSE28007)/Homer | 1e-2 | -4.611e+00 | 0.0527 | 18.0 | 1.80% | 459.1 | 0.96% | motif file (matrix) | svg |
| 84 | T A C G T C A G T G C A A G C T T G A C A G C T A G T C A C T G G A T C A C T G T C G A A C T G C T G A C T G A A T G C | ZBTB33(Zf)/GM12878-ZBTB33-ChIP-Seq(GSE32465)/Homer | 1e-2 | -4.605e+00 | 0.0527 | 10.0 | 1.00% | 197.7 | 0.42% | motif file (matrix) | svg |
